# Editorial: Insights in tissue engineering and regenerative medicine 2021: Novel developments, current challenges, and future perspectives

**DOI:** 10.3389/fbioe.2022.1125027

**Published:** 2023-01-12

**Authors:** Ranieri Cancedda, J. Mary Murphy, Martijn Van Griensven

**Affiliations:** ^1^ University of Genova, Genova, Italy; ^2^ Regenerative Medicine Institute, University of Galway, Galway, Ireland; ^3^ MERLN Institute for Technology-Inspired Regenerative Medicine, Maastricht University, Maastricht, Netherlands

**Keywords:** tissue engineering and regenerative medicine, stem cells, biomaterials, Platelet Rich Plasma, organ-on-a-chip, clinical trials

The Tissue Engineering and Regenerative Medicine (TERM) pursues a multidisciplinary approach to the development and application of new therapeutic strategies and products for the treatment and repair of tissue defects and disease modulation. Starting at the end of last century, major progress has been made in this field and, especially in the last years, the results obtained by the TERM scientists have been exceptional. This Research Topic outlines recent developments, major achieved accomplishments, and future challenges. The original articles and the reviews highlight some of the latest advancements at the forefront of different aspects of TERM. Hopefully, this article Research Topic will inspire, inform, and provide direction and guidance to a new generation of TERM researchers.

Stem/progenitor cells are key elements in TERM. Among them, the more frequently adopted are Mesenchymal Stem Cells (MSC) from different tissues. The review by Al-Ghadban et al. describes current clinical applications of adipose derived MSC and looks forward to future applications of these cells, including the development of organoids, tissue elements, and organ-on-a-chip systems. Due to the context-sensitivity of MSC to their microenvironment, it is evident that the therapeutic efficacy of these cells is modulated by the pathologically altered tissue environment. Questioning whether the context-sensitive interaction between MSC and specific targets leads to an enhancement, or an inhibition of the therapeutic effects is of crucial significance. The review by Roth et al. presents the author’s viewpoint on pathology-related targets of MSC therapeutically applied in tendon and joint diseases, focusing on the equine patient as a valid animal model.

Major progress was made with the transplant of “*ex vivo*” expanded autologous (from the patient) stem/progenitor cells seeded on or associated to carrier biomaterials. The purpose of the review by Selvakumar et al. is to explore the application of regenerative medicine principles into current and future stent designs. The review covers regeneration-relevant approaches emerging in current research and highlights two unique regenerative features of stent technologies: selective regeneration, and stent-assisted regeneration of ischemic tissue as consequence of an injury. When cells are implanted in association with biomaterials and/or medical devices some weaknesses still hinder the TERM approach. The analysis performed by Adamo et al. proposes a critical overview to identify key aspects to be considered and implemented in designing new tracheal substitutes, thus paving the way towards safer and more effective solutions for treating patients, now incurable.

The increasing knowledge about the physiological body response to injury suggests that the human organism itself could provide all elements needed for tissue repair and regeneration. New strategies, aimed at the stimulation of the body resident stem cells and of the intrinsic endogenous potential of tissues to heal or regenerate, are being developed. Lower limb ulcers represent a major clinical problem for the aging human population, and particularly for diabetic patients. More recently, treatments with allogeneic stem/progenitor cells, cell released microvesicles, or with platelet derivatives, such as Platelet Rich Plasma (PRP), have been proposed as alternative therapies for chronic skin ulcers. In most cases, this resulted in a significant benefit for the patient. The review by Mastrogiacomo et al. summarizes results obtained with these innovative approaches for the treatment of diabetic ulcer patients. The physiological body response to an injury includes major changes in key signaling molecules, such as Reactive Oxygen Species (ROS), that play an important role in the progression of inflammatory disorders. Our understanding of the effects of an enhanced ROS generation on cellular processes has been largely established, but their therapeutic potential is mostly unexplored. The manuscript by Sheppard et al. provides a view of the effects of ROS on skeletal healing. Hopefully, this will allow development of novel strategies to optimize the redox environment for skeletal tissue regeneration.

Advances in imaging techniques are crucial in the progress of the TERM field. The review by Huang et al. is focused on the application of nerve tracer imaging and summarizes current knowledge and mechanisms of action regarding nerve regeneration in organs where transplantation techniques have been widely performed, such as heart, liver, and kidney.

The adoption of suitable preclinical models is a must before considering any human treatment. Given the increasing demand for animal free alternatives in biomedical research, the manuscript by Munzebrock et al. reviews the applicability of some *in vitro* models to mimic the osteoarthritic (OA) joint, focussing on the crosstalk between the different joint tissues. In several of the described models a response to stimuli or drug treatments was observed that mimicked OA *in vivo* processes. In the same line of research, the review by Mainardi et al. analyses existing organs-on-chip platforms used to investigate pathological alterations of intervertebral discs (IVD). The article also proposes the conceptualization of a prospective IVD-on-chip model that could be used for mechano-transduction studies and therapy testing.

Present regulatory laws and ethical concerns recommend that controlled randomized clinical trials be performed before a new tissue engineering and regenerative medicine strategy or tool can be transferred to routine clinical practice and adopted for many patients. Clinical trials with regenerative therapies have shown some limitations, challenges, and uncertainties. However, the field’s potential and implications remain great. The review by Petrosyan et al. is an overview of current TERM clinical trials and highlights how regenerative medicine aims to deliver effective patient-specific treatments with lifelong benefits. Looking to the future, the manuscript of Petrosyan et al. presents an update on the recent advances in transplantation that, by combing transplant and regenerative medicine fields, may change the way we think and practice tissue and organ transplantation.

While tissue engineering and regenerative medicine has been progressing over the past two decades, the sub-field of regenerative endodontics is one of the few areas of the field with true application that is currently being implemented in everyday clinical practice. The so called “Minimally Invasive Endodontics” has the goal of conserving tooth structure during conventional root canal therapy to enhance tooth integrity and survival thus addressing naturally inducing regeneration of what has been lost (Elnawam et al.).

Although, in principle, the whole world’s population could benefit from these new technologies, at present there are very significant differences in their adoption in different regions and countries of the planet. To fill the existing gaps, specific strategies could be adopted by governments. The study by Kim and Bae reviews the change made by the Advanced Regenerative Medicine and Advanced Biological products act (ARMAB) implemented by the government in South Korea in 2020. We believe that this example could make a significant contribution to other countries which have plans to promote regenerative medicine.

